# Unifying to Advance Understanding: Collaborative, Community-Driven and ‘Open’ Approaches for Better Science in Sport

**DOI:** 10.1007/s40279-026-02394-8

**Published:** 2026-03-16

**Authors:** John Warmenhoven, Ian Shrier, Garrett S. Bullock, Alex O. Holcombe, Sophia Nimphius, Paolo Menaspà, Aaron J. Coutts, Franco M. Impellizzeri

**Affiliations:** 1 School of Sport, Exercise and Rehabilitation, Faculty of Health, University of Technology Sydney (UTS), Sydney, Australia; 2 Human Performance Research Centre, University of Technology Sydney (UTS), Sydney, Australia; 3 Australian Sports Commission, Australian Institute of Sport, Canberra, Australia; 4 Centre for Clinical Epidemiology, Lady Davis Institute, Jewish General Hospital, McGill University, Montreal, Canada; 5 Department of Orthopaedic Surgery & Rehabilitation, Wake Forest University School of Medicine, Winston-Salem, USA; 6 School of Psychology, University of Sydney, Sydney, Australia; 7 School of Medical and Health Sciences, Edith Cowan University, Joondalup, Australia

## Abstract

Historically, sport science and medicine researchers have had difficulty obtaining adequate sample sizes, limiting their ability to make accurate generalizations to populations of athletes. To move towards better and more generalizable research, sport could look to other fields of research, and draw upon collaborative approaches for performing science. These include concepts such as big teams approaches for conducting experimental research, developing data repositories centred on a holistic inter-disciplinary view of the athlete and exploring privacy preserving meta-analytic inspired concepts such as federated analyses or pooled synthetic data. These approaches have enormous potential but do come with their own difficulties and risks. There are challenges surrounding the collection, ownership and perception of data, particularly in high-performance sport contexts. This conceptual review is a step towards presenting possible solutions, which could be tested, documented and sculpted to fit the high-performance and professional sport environment, with the aim of improving the processes underpinning scientific enquiry in sport.

**Supplementary Information:**

The online version contains supplementary material available at 10.1007/s40279-026-02394-8.

## Key Points

**Table:** 

Collaborative science for sport will lead to:
1. Better-informed judgements for groups that have either very low samples (i.e. paralympic and elite world class) or samples that may be difficult to access due to organizational or league-related restrictions.
2. Better understanding of whether effects generalize across factors of interest, inclusive of (but not limited to) coaching environments, geographical locations, other socio-demographic characteristics, athlete training and injury histories and pathway trajectories into sport.
3. Better understanding of why concepts may not generalize for specific sub-groups in high-performance sport populations. For example, in paralympic sport, generalizing across class and impairment type may be difficult due to the variability within classes. Collaborative approaches have the potential to uncover sub-groups that are different, and this can be used to frame new hypotheses and exploratory research to understand why this may be the case.

## Introduction

The replication crisis has affected many scientific fields, including medicine, economics and psychology [1]. Recently, an investigation of the replicability of applied sports and exercise science research showed that fewer than 30% of the investigated studies could be replicated [2]. Additionally, within sport, several studies have alluded to methodological issues, which could act as the first warning signs that particular areas within sport research are facing replicability problems. For example, between 70 and 82% of published studies in sports science and medicine journals reported significant findings [3, 4], with similarly high levels of significant findings being identified in the reporting of sports and exercise medicine journals [5, 6]. It is unlikely that the high proportion of significant findings noted in sport research are due solely to high-quality research designs and testing true effects [6]; rather it may be attributed in part to publication bias (and the file-drawer problem) and questionable research practices (QRPs) (including HARKing and *p*-hacking) resulting in an artificially high proportion of significant findings [6].

Additionally, sport research likely suffers from issues related to underpowered study designs [6, 7]. This problem is common in several fields such as psychology, where 90% of published studies reported significant findings, but the average power to detect a medium effect size has been estimated to be less than 50% [8, 9]. The low quality of power analyses has been highlighted as a concern in sport science research [10] and a recent study by Mesquida [11] reported that the average power of studies in sports science is as low as 11% for detecting a small–moderate effect. Suboptimal sample sizes increase the risk of failing to detect an effect of relevant size [6] and the chance of detecting a true effect. In a context with publication bias, low statical power reduces the likelihood that a published statistically significant result reflects a true effect, because smaller sample sizes can produce exaggerated or smaller effects due to sampling variability, where only extreme effects will reach significance. In the absence of publication bias, the pooled effect size estimate would remain reasonably accurate even with small samples. However, it is the presence of publication bias and/or questionable research practices in combination with low statistical power that distorts the literature and leads to inflated effect size estimates. Given that these issues are present in fields such as psychology, a similar ‘crisis in confidence’ is also likely beginning to grow in parallel, within sport science. Although not a justification for the widespread use of small samples, it should be acknowledged that studies conducted in elite (or high-performance) sport contexts have the additional limitation of smaller athlete pools [12–14]. This is exacerbated in the paralympic sport context, where there are ten impairment types within each sport, with the potential to result in smaller and heterogeneous populations of ‘para’ athletes within research studies [15].

The potential barriers to collecting a sufficiently large sample to have adequate statistical power is also a challenge in certain areas of medical research [16–19]. In addition to the difficulties in obtaining representative samples, recent reviews of athlete monitoring and surveillance systems have identified issues with reporting of the data quality (i.e. completeness and validity) [20], further contributing to issues in conducting generalizable research. Ekegren et al. [19] noted that many existing systems lacked validation against independent data sources, suffered from inconsistent injury definitions, incomplete reporting fields, and data entry errors, each of which undermines the reliability and comparability of surveillance datasets. Additionally, data that are complete, or accurate, and retained for analysis may not be representative of the athlete cohorts that are being analysed, further highlighting issues with conducting generalizable research. In addition to issues with smaller samples and under-powered studies, there are further demonstrated issues in the conduct of research in sports science and medicine, including problems with the transparency, reproducibility and methodological rigor in meta-analyses [21] and issues in the appropriate construction of hypotheses and application of appropriate statistical tests for testing these hypotheses [22].

The purpose of this article is to offer practical suggestions and potential solutions for the sports science and medicine communities to consider in the conduct of research, particularly for difficult-to-access populations, such as elite athletes, whilst still maintaining and preserving high research quality. These suggestions are borrowed from other areas that have already taken steps to address the challenges and includes researcher- (or people-) driven initiatives such as ‘big team’ approaches to science, and data-centric approaches, such as data sharing, big data repositories and federated data models.

## Big Team Science

‘Big team science’ generally relates to collaborations where a large number of researchers pool their expertise and resources in pursuit of a common objective or goal [23]. Collaborators within big teams can be dispersed across industries, institutions, disciplines, cultures and continents. This differs from conventional approaches to scientific research, where activity is typically organized around smaller research groups or single principal investigators [24, 25]. Examples of big team science are established across other fields [26–28], where many universities and research institutions have conducted research collectively through the centralization of resources, development of processes for developing meaningful research questions and standardization of appropriate analytical methods used within specific fields [23]. A big team approach allows specific researchers to specialize into roles that match their skills rather than being required to fulfil all roles simultaneously (i.e. experimental design expertise, data collection expertise, statistical expertise, etc.) [24], and also provides a valuable mechanism for creating inter-disciplinary teams of researchers and scientists, ensuring that the correct expertise is present for a given project.

A major benefit of big team science is the development of a community. Researchers who join a big team science group gain access to a network of researchers, reducing intellectual isolation for individuals and generating new collaborations that can bring impact to the broader sporting community. In sport, the inclusion of inter-disciplinary expertise within a big team approach to science could facilitate the development of methods and infrastructure that are appropriate for the context of high-performance sport. As an example, the inclusion of statistical expertise in a big team approach would help avoid study design errors, whilst also facilitating use of the best statistical models for the problem [29]. For the practicalities of developing a big team, there are several initiatives in other fields that sport can learn from, some of which are covered in the following section (and in Supplementary 1 Table). These examples show an improved ability to generalize across contexts (i.e. culture and geography), build infrastructure for measurement and testing that is consistent and reproducible across testing sites and adopt a community approach to developing and testing theories, contributing to meaningful research designs and moving towards a systemic approach for coordination in science [30].

### Generalizing across Contexts: the Example of the Psychological Science Accelerator (PSA)

The Psychological Science Accelerator (PSA) was born out of the *ManyLabs Replication Project* [31], which was an initiative designed to assess the replicability and generalizability of psychological science. The PSA has demonstrated a unique ability to test generalizable concepts across different contexts, including (but not limited to) cultures and geographical locations. Examples of these projects include studies focussed on ways of communicating to motivate social distancing in the context of the coronavirus disease 2019 (COVID-19) pandemic [32], and cultural factors that affect moral judgements [33]. These processes investigate whether different experimental effects hold across geography and culture. Such goals are also relevant for sport, particularly for evaluating generalizable effects across different training environments and locations. As an example, when evaluating specific skill acquisition interventions in the context of paralympic research, substantial differences may exist in the form of coaching philosophies, training cultures and athlete histories. As noted by Askew et al., [34], from the perspective of coaching in paralympic sport, there can be a reduction of formal developmental opportunities for coaches [35], leading to coaches learning via varied (non-formal and informal) experiences [36], potentially yielding greater variability in coaching philosophy and design of training for athletes. A big team approach may be necessary for understanding whether specific effects hold for paralympic athletes across these varied training and coaching environments.

It is important to acknowledge that the pursuit of generalization in sport could oversimplify the socio-cultural factors that shape training and coaching practices. Our intention is not to claim that big team science could flatten these complexities in sport, but rather that larger-scale, multi-site collaborations offer a framework for systematically identifying where generalization is possible, and where contextual constraints resist it. For example, a big team approach could empirically test whether certain physiological training adaptations are consistent across diverse contexts, whilst simultaneously revealing cultural or contextual differences in how training is delivered and experienced. In this sense, generalization does not imply erasure of socio-cultural nuance; instead, it can highlight the boundaries of applicability and direct attention to precisely those areas where contextualized, theory-driven explanations are required. Thus, big team science is not a substitute for metatheoretical reflection, but a means of empirically mapping both commonalities and divergences across sporting contexts.

### Infrastructure for Continual Data Capture: the Example of the Disturbance and Resources Across Global Grasslands (DRAGNet) Network

DRAGNet is an example of a research network that provides infrastructure for continual data capture. Developed out of the Nutrient Network, it is focussed on understanding how disturbances and nutrient inputs affect the recovery and assembly of plant communities in grasslands around the world. Data synthesis and database compilation occur as part of project research, and these databases offer unique opportunities for collecting data across different timescales and geographical locations.

In sport, building robust data capture and storage infrastructure could facilitate the management of continual streams of data, allowing for analysis across longer time scales. Athlete monitoring over time should yield improved decision-making in high-performance sport environments [37, 38]. By extending analyses across entire years/seasons, this type of infrastructure creates possibilities for understanding whether effects of interest hold across timepoints relative to periodized training programs. A big team approach may also be required for developing and building such repositories, given that athletes may be connected to different sporting systems and training programs.

### Meaningful Data Collection and Testing: the Example of Many Babies and Many Primates

*ManyBabies* [27] and *ManyPrimates* [28] are two collaborative initiatives for best scientific practices on subject groups that can be difficult to analyse. In *ManyBabies*, questions are tested across laboratories, methods and populations, assessing the generalizability and robustness of outcomes across different contexts. Although high-performance sport is vastly different to both research with infants and primate cognition research, there are some similarities with the challenges that high-performance sport face with small populations. ManyBabies highlights the value of using a big team community for research questions that have a strong theoretical or conceptual foundation to minimize the likelihood of research waste [39]. Sport could benefit from a similarly careful and considered approach to the generation and prioritization of research questions within a big team approach. Additionally, sport science could adapt the *ManyPrimates* approach to making inferences. In part, the approach makes inferences about a given species of primate’s behaviour on the basis of other closely related species. Similarly, sport could gather preliminary evidence across athlete groups that are easier to access and measure as an avenue to better tailor and develop interventions for athlete groups that might be more difficult to access or sparser in number. This could include leveraging information from more common paralympic classifications to inform initial interventions for classifications that are rarer, or undertaking a similar process for informing interventions for rare medical or injury conditions. The IOC consensus on recording and reporting of data for injury and illness for para sport [40] draws attention to this issue for para athletes, where for injury surveillance purposes, para athletes must be grouped in a pragmatic way given the inherently small samples in some para and medical classifications.

### Big Team Science: Considerations

Initiatives such as the PSA, NutrientNetwork, ManyPrimates and ManyBabies provide examples of the possibilities for big teams approaches that could be developed in sport. To realize the possibilities for sport, however, there are a number of factors to consider.

#### Infrastructure

Digital infrastructure is essential in big teams for facilitation and coordination of projects [24]. Platforms such as Slack, the Google suite of collaboration tools and Zoom have been mentioned as avenues for team communication and document management in big team science [26]. A big team approach in sport will need to find software that matches university, institute or organizational infrastructure involved in the research, given that communication between team members may be restricted to specific platforms that are approved by information and communication technology (ICT) governance structures, as well as specific data governance policies (if communication channels also involve transfer of data). These types of software and platforms will need to be compliant with relevant ICT and data governance policies at sporting institutes and government organizations (which may handle the data collection of high-performance athletes). This may be problematic for more ‘open’ tools (i.e. Google suite of tools, Slack, etc.) and may require the development of systems that are compliant with the specific security requirements and needs of sport. Examples of bespoke solutions have been developed for other collaborative science initiatives, such as the *formr* experiment platform [41] for survey-based research. This consists of simpler survey frameworks (i.e. Google sheets), a study control framework (for managing administrative and communications related components of research projects) and an R package for cleaning and combining data across collection points. Similarly, conventions such as the Brain Imaging Data Structure (BIDS) have been designed in neuroimaging experiments, serving as a standard for organizing and describing magnetic resonance imaging (MRI) datasets whilst also unifying practices that are already common in neuroimaging research [42]. In sport, similar conventions might become important for the standardization of collection and data storage processes for motion capture data in sports biomechanics, where different systems, models and marker sets may be used, and also for sensor technology more generally in athlete monitoring.

Accessibility to software and platforms (and some level of ‘openness’) in big team science is important, particularly in cross-university and cross-country collaboration. Some individual university/organizations may not have a subscription that allows external collaborators to connect in. Additionally, the cost associated with adding external users through off-the-shelf products may prohibit different groups from becoming involved with a big team. Ultimately, adherence to ICT and data policies are essential for considering big team science initiatives in high-performance athlete data, and additional considerations around cost and accessibility are important for developing a big team process that is feasible for sport. These costs have to be weighed against the wasted resources associated with underpowered studies, inability to generalize results to specific sports and the biases that result when small research teams do not have personnel with all the necessary expertise.

#### Project Management and Leadership

From an operational perspective, large-scale collaborations require project management for coordination and planning and building the foundations for effective governance. Project management software is ubiquitous to that used outside of the academic sector, but often is not required or drawn upon in academic science. Some early stage project management software exists (see STAPLE at https://staple.science/) that can help track data and metadata. This would, however, require careful thought for integration into the high-performance sport system, where entities such as universities, sport institutes and national governing bodies need to be considered.

When built on strong foundations of project management, efficient and effective project coordination becomes possible within a big team initiative. This is an important consideration for sport, given that researchers may not have the skills or education in project management. In light of this, successful management would need to encompass effective coordination of face-to-face or web-based meetings, documenting outcomes from meetings and leading discussions between participating labs across different project phases, data validation and quality checking, as well as analysis, writing and manuscript revision. Byers-Heinlein et al. [43] suggest a potential benefit of having two different types of project leads: (1) an administrative lead (e.g. a research manager) to facilitate meetings, coordinate dissemination of documents and information between participating labs and facilitate project implementation; and (2) scientific leadership (typically a small group of leads) to focus on question formulation, experimental characteristics (design, analysis, etc.) and article writing [43].

When looking more deeply at the importance of scientific leadership, ManyBabies leadership teams are formed around specific projects (with these teams being named sequentially, that is, ManyBabies 1, ManyBabies 2, etc.). Each project-based leadership team plays a critical role in ensuring that diverse views are heard, whilst discussions are directed towards productive outcomes [43]. In addition, as ManyBabies has grown, community-level leadership has also been established (i.e. a governing board), which is responsible for ensuring the activity of ManyBabies conforms to its vision, and that research successes are retained within the big team [43]. Forscher et al. has also alluded to the risk of unaccountable leaders [24], with it being essential that big team science initiatives construct bylaws that lay out the distribution of power, responsibility and accountability for making decisions within the big team community. This is important when considering the context of high-performance sport environments, where a significant burden may be placed on non-researching scientists and practitioners who are embedded in institutes of sport, national governing bodies or professional teams and associations.

#### Governance and Documentation

Governance and documentation follow from management and leadership and underpin many of the principles enforced by leadership groups in big team communities. Governance and documentation are essential for conflict avoidance in the context of issues related to timelines and disagreements over research objects (e.g. publications and data). Baumgartner et al. [44] has noted the importance of ‘collaboration agreements’ in big team science to provide all parties with clarity and transparency about project goals, expectations and policies [42]. These agreements cover definitions (and responsibilities) within roles, authorship criteria, processes for conflict resolution and expectations for project deliverables [44]. Baumgartner et al. also acknowledged the risk that collaboration agreements could be viewed as aversive for new researchers who are new to a big team. They recommended that agreements focus on what specific agreement detail adds to a project (e.g. transparency, accessibility) rather than what it prevents (e.g. conflict, authorship disputes) [44].

Issues related to governance also extend to the management of data collected within big team projects. Particularly within sport, big teams must put processes and documentation in place that can navigate specific data governance constraints. As a broad example, the General Data Protection Regulation (GDPR) law enacted by the European Union imposes rules for collecting, storing and sharing personal data [44, 45], affecting what ‘can’ and ‘can’t’ be done with data belonging to European athletes. Similarly, at the organization, club or league level, data may be viewed as a commodity with competitive advantage, and sporting organizations may have their own policies around data protection (see Sect. 3 for more details). To avoid such issues, many big team initiatives in other fields opt to make the collection of sensitive or controlled data optional across sites unless it is absolutely necessary for the research question of interest [44].

This also extends to issues in the processes and management of data. Some important considerations here relate to the validation and accuracy checking of data, as it is coming from multiple sources into big team infrastructure. ManyBabies [43] cited this as a potential issue for their own projects within the big team approach, with there being issues around data validation and processing relative to the software and code that had been developed for analysis. There were challenges related to processing and merging the datasets after collection, despite having data formatting templates with definitions of each variable [43]. For sport, training and specialization will become integral for addressing the risks associated with data processing and management across testing sites. Strategies such as standardizing data collection procedures, using consistent measures and implementing standardized pipelines for cleaning, processing and analysis could mitigate these problems.

#### Authorship Issues and Contributors

Rewarding team players in big team initiatives can be difficult due to misalignment with university incentive structures (i.e. big teams lead to a smaller number of publications that take longer to collect data for and publish) [23]. Aligning incentive structures can also be difficult for scientists who work in sport (i.e. non-academic staff of sporting organizations), with these activities not being a regular part of the servicing that they provide. ManyBabies acknowledged that whilst aiming to be inclusive across different research groups, it was important to avoid authorship ‘padding’ on papers [43]. To mitigate against this, ManyBabies limited individual labs whose main role was data collection to two authors (i.e. the principal investigator and a junior researcher), with there being some flexibility for deviating away from this under specific circumstances [43].

Another way to mitigate against this particular issue would be to re-define how contributors to a big team approach are acknowledged. ManyPrimates [28] noted the various ways research contributions take place within a big team, including facilitating and coordinating discussions of study topics, writing protocols, developing code, and curating and analysing data and manuscript preparation. ManyPrimates have specified authorship guidelines (see: https://manyprimates.github.io/authorship/) to clarify the different ways that contributors can gain authorship on project publications [28]. In addition, Holcombe [46] has drawn attention to the need to better credit the contributors to research outputs in the context of authorship guidelines that prevent specialists who do not contribute to the writing (common in big team science) from becoming authors. There is a need for publishers and societies to build upon initiatives such as Contributor Roles Taxonomy (CRediT) to move towards changing the criteria for authorship, particularly as big team projects become more common with larger numbers of contributors [46]. This includes using more appropriate frameworks to define contributions, such as the CRediT framework for standardizing research contributions and increasing transparency (http://www.casrai.org/credit.html) [47].

#### Unifying Principles in Big Team Science

A question for sport might be how a big team would come together. Initiatives in other fields illustrate several pathways. Some networks, such as the Psychological Science Accelerator, are united by a metatheoretical commitment to cumulative, replicable science, and then democratically select empirical questions that align with that vision. Others, such as DRAGNet or ManyBabies, coalesce around a specific class of research questions, drawing in diverse expertise as needed. In sport, both routes have merit. A common metatheoretical stance, such as improving the generalizability and robustness of sport science, could provide long-term connection for a community, whilst specific research questions (e.g. injury surveillance in paralympic athletes, or multi-year athlete monitoring) would mobilize the interdisciplinary skills required for individual projects. Thus, big team science in sport may benefit from a hybrid approach: a shared overarching vision, with flexible project-based collaborations that evolve according to the questions being addressed.

#### Breadth of Expertise in Big Team Science

Whilst much of our discussion has emphasized data-related challenges of sport science, we recognize that true big team approaches must extend beyond quantitative expertise. The value of big team science lies not just in scale but in breadth of perspectives, including expertise from philosophy, ethics, qualitative social science and cultural studies, which would broaden the types of questions asked, as well as how those questions are framed. For example, qualitative researchers could reveal contextual, experiential or cultural insights that are often overlooked in strictly quantitative designs. Such inclusivity is particularly important in sport, where the lived experiences of athletes, coaches and communities interact with performance and health outcomes in ways not easily reduced into quantitative data. Thus, in sport, ‘big team’ must be understood not only as a larger collaboration, but also as broader in disciplinary scope, integrating diverse methodological and theoretical traditions alongside data-centric expertise. Similarly, big team science requires expertise in project management, ethics and stakeholder engagement. Thus, the expertise gap is not simply technical but also conceptual and relational, underscoring the need for deliberately broad and inclusive team composition.

#### Clarifying Collaboration versus Big Team Science

It should be acknowledged that ‘collaborative science’ is a broader category than ‘big team science’, and that the two are not interchangeable. Collaboration in sport can take many valuable forms, such as thematic research programs, cross-departmental organizational structures that encourage dialogue across disciplines or systems-level inquiry focussed on organism–environment interactions. Big team science is one particular model of collaboration: large, multi-site, multi-disciplinary teams working under shared structures of governance, with specific mechanisms for data pooling, coordination and replication. Whilst the current commentary has focussed on this model, it should not be viewed as the only or necessarily the universal approach for conducting collaborative science. Rather, big team science should be viewed as complementary to other collaborative strategies, particularly suited to questions that demand large samples, cross-contextual generalization or methodological standardization.

Despite this, collaboration is still central to successful big team approaches, with successful collaboration requiring not only accessibility to software and platforms (as already mentioned), but also a willingness to share knowledge and infrastructure. Additionally, it requires adjusting individual research strategies to align with shared goals. Achieving meaningful progress requires better training and education so that researchers and scientists in sport better understand the limitations of isolated work and the benefits of collaboration over competing for scarce resources. Big team approaches provide an opportunity to achieve this progress within sport.

## Data-Centric Approaches

The previous sections on big team science have focussed on the people element of research (i.e. building teams, networks and communities). Not completely independent from this are data-centric approaches for collaborative science, with these ideas being potentially complementary to big team initiatives. Combining data within a larger multi-group research initiative requires standards for capture of information. Developing standards for consistent and quality data capture is something that has long been of interest to sport. This includes having well-defined injury surveillance and monitoring systems in high-performance sport contexts (please see [20] for a review of 15 different databases for injury surveillance) and established standards for recording and reporting of epidemiological data on injury and illness in sports [48]. In the domain of sport science, national institutes such as the Australian Institute of Sport have developed schemes such as the National Sport Science Quality Assurance (NSSQA) program for quality management of data [49]. The NSSQA standards were based on the ISO-9001 standards^1^ for ‘quality management systems’ but retained the components of relevance to areas such as exercise physiology [49]. Additionally, at the research community level, the sports biomechanics community has demonstrated progress towards standardizing data collection processes between labs to improve the ability to build larger evidence bases [50, 51]. Approaches for improving accessibility to data for collaborative research often centre around data repositories. These repositories can be small, in the sense that the data being stored within them occurs at the level of a specific project, or these repositories can be large, with ongoing data being collected and continually added to the repository, with researchers being able to analyse large amounts of interconnected variables. Both of these will be discussed within the context of sport research.

### Data Sharing Methods

Currently, many repositories are set up to enable data accessibility alongside published research [52] to promote data sharing and availability. Data are often shared as a part of a ‘self-deposit’ model, where a publisher recommends online platforms where data can be made open and accessible. In one example, initially highlighted by Austin et al. [52], the journal *PLoS One* listed 7 cross-disciplinary repositories, as well as 91 repositories which were specific to 12 different categories of research area: unstructured (and/or large) data; sequencing; omics; structural databases; neuroscience; model organisms; taxonomic and species diversity; biomedical sciences; biochemistry; physical sciences; social sciences; and marine sciences [53]. These repositories house data at the level of specific projects, making it difficult to combine datasets for common research questions, given that the data are not necessarily captured as a part of the same protocol. Nevertheless, these data are open and accessible, and if a new group wants to reproduce outcomes from these studies it is possible to do so matching the exact composition of the original dataset.

There are incredibly important considerations about feasibility for regular repositories that facilitate the ability to perform secondary analysis of stored data. Firstly, there is a need for quality metadata. Metadata capture the essential contextual information (i.e. who, what, when, where and how about a dataset), supporting its findability, accessibility, interoperability and reusability (FAIR) within research data repositories [54]. In a review of the minimum requirements for data repositories, Austin et al. [52] found that the support for standard metadata was highly variable between the 32 repositories and data sharing platforms evaluated. More than two-thirds of the repositories provided some support for metadata creation (i.e. guidelines, templates, review, etc.), but the majority of larger repositories left metadata quality control to the data providers. This is problematic, given that metadata are essential for ensuring that the data are correctly understood. Additionally, for more difficult to access groups of athletes, these regular repositories could form an initial step towards building larger volumes of information and more robust levels of evidence for questions related to groups that have smaller populations. Well-collected datasets that form smaller open repositories also make it more appealing for quantitative disciplines (i.e. statistics and data-science) to collaborate with sport researchers and scientists. Sport could look to adopt a similar approach to social psychology, which has taken a community-driven approach for the development of Psych-DS (https://psych-ds.github.io). This is a community data standard specification that provides a systematic approach to organizing scientific datasets, including metadata, so that data are shared in a machine-readable format and can be easily reused in the social sciences.

### Big Repositories

Standardized repositories often represent a valuable first step and could be expanded to house larger volumes of data relative to sport-specific research questions, but these tend to be ‘bottom-up’ agreements amongst researchers to deposit somewhat-disparate data into repository. This is less than ideal for sport, given that each of the deposited datasets are stored independently and not necessarily architected for integration with other data sources. Similarly, the quality of the curation of these repositories lies in the hands of those uploading the data, and there may be issues ensuring enough information is provided in the form of metadata to enable secondary exploration of these data. This speaks to the need for something that is more robust.

‘Big data repositories’ (our term) are systematic, prospective collections of data that aim to avoid many of the issues common to standard project-specific repositories, such as inconsistent data collection methods, variable metadata quality, fragmented datasets and limited long-term sustainability. The United Kingdom BioBank (UKBB) (https://www.ukbiobank.ac.uk/) is probably the most well known, and is a large-scale biomedical database and research resource containing genetic and health information from more than 500,000 UK participants. This initiative has led to more frequent and sustainable research in the space of biomarker and phenotypic data, with Huang et al. [55] noting that from January 2019 to November 2020 there were 1432 PubMed-indexed publications citing the ‘UK Biobank’ or ‘UKBB’. The UK Biobank operates as a centralized research resource in which participant data are collected prospectively through standardized protocols by accredited personnel, securely linked to health records and made available to approved researchers under regulated data-access agreements. This approach requires a substantial amount of sustained funding.

In an area of genomics called genome-wide association studies (GWAS) [56], researchers test hundreds of thousands of genetic variants across large numbers of genomes to identify those that are linked with a specific trait or disease. GWAS studies leverage computerized databases containing data related to the human genome sequence, maps of human genetic variation and advanced technological applications for analysing whole-genome samples [57]. GWAS studies have led to the National Centre for Biotechnology Information (NCBI) developing large databases for use by the research community [57]. Additionally, archives of GWAS data can be accessed through the Database of Genotype and Phenotype (dbGaP) website (http://www.ncbi.nlm.nih.gov/entrez/query.fcgi?db=gap). This approach has succeeded because genomic data has become highly standardized.

Centralizing sports data in similar big repositories would more broadly allow for important questions to be answered about very specific and difficult-to-measure populations in sport and provide interesting data contexts for researchers in statistics and data-science to connect with for progression of their own theory and methods. Similar efforts as those required for big team science approaches would be required from the sport community for architecting, housing and maintaining the infrastructure necessary for a big sport data repository. Additionally, care and consideration would be required in the development of standards for data being entered into such a repository.

### Considerations for Data-Centric Approaches

Despite the potential benefit of big repositories providing the possible ‘scaffolding’ for collecting data systematically in sport, and the potential of such repositories for facilitating sustainable research practices in sport, there are potential considerations for designing and using these repositories that must be acknowledged.

#### Facilitation of Potential Selection Bias and Poor ‘Representativeness’

Firstly, unlike big team approaches, these big data repositories do not necessarily require for a question to be framed prior to prospective collection of data. The power of these repositories lies in the breadth of information that is housed within them at the level of the individual. For example, the UKBB houses information on more than 500,000 people inclusive of neurological, cardiac and MRI imaging, dual-energy X-ray absorptiometry (DEXA) scans, ultrasound data, genotyping data, and connections to health records (e.g. electronic health-related records related to mortality, cancer, hospital admissions and primary care records), as well as blood and urine samples and responses to various online questionnaires [58].

Conceptually, this could be viewed similarly to obtaining data from routine testing within each discipline of sport science and sports medicine, for as many athletes as possible, making it possible to perform valid explorations and frame hypotheses for questions that sit across discipline boundaries. It sounds wonderful, but even this comes with challenges. Using the UKBB as an example, Huang et al. [55] highlighted that since its inception in 2012, quantitative researchers (i.e. epidemiologists and biostatisticians) have raised concerns about inferential challenges presented by the convenience sampling presented in the UKBB [59, 60]. These inferential challenges stem largely from the non-random, volunteer-based recruitment of UK Biobank respondents, which can produce systematic differences between participants and the general population. These concerns would apply equally to any voluntary-participation big data resource, where many unknown, and potentially unknowable, selection mechanisms have the potential to influence statistical inference [60]. In practice, this means that individuals who choose to participate in research may differ systematically from those who do not. In a sport context, this might occur if athletes from well-resourced programs are over-represented in a large repository, leading to estimates of training load, injury risk or performance relationships that may not generalize to athletes in less-resourced environments.

#### Data Administration and Governance

In addition to potential selection bias issues mentioned above, an additional consideration for big data repositories is who is responsible for the data curation. Data curation differs from data quality, in that it relates to the activity of managing data to ensure that the data are fit for contemporary purposes and available for discovery and reuse [52]. For surveillance programs, this means continuous enrichment and updating to maintain recency for new and evolving research purposes and questions. This will inevitably place a substantial administrative burden on someone (i.e., an individual or group) to maintain and curate the database. This includes decision-making about which data are retained in the repository, the duration and conditions of their storage and the procedures used to review, validate and maintain data quality over time.

There is also a need for consideration around governance processes for how ideas are decided upon for experimental research themes, or specific data contexts for repositories. This could be done through formalized processes, such as voting on ideas at a society or network level, or moving towards the use of formal frameworks for setting research priorities such as those developed for population health and medicine [61, 62]. There must also be consideration around how additive ideas are included within any initiative (i.e. the development of protocols for add on experiments or new data contexts beyond the scope of the initial launch) to ensure that it is still possible to mitigate against potentially questionable research practices.

#### Privacy and Anonymity

Privacy and anonymity issues with repository data are especially important in high-performance contexts where third-party reports of some injuries or performances may allow for identification of participants. This could be due to a number of factors, including the sensitivity of the information being collected around athletes (i.e. medical and health information). There are some potential alternatives for handling the issue of making individual observational-level data open, which are derived from fields such as medicine, epidemiology, statistics and data science.

##### Data Warehouses with Gate-Keeping

Data warehouses with gate-keeping are centralized encrypted databases. After researchers submit a research proposal, a reviewing committee accepts or declines the data query request [63, 64] (e.g. ClinicalStudyDataRequest.com). This provides better protection of patient data privacy whilst maintaining data availability. There are some limitations, including only being available to researchers who meet eligibility criteria for access (i.e. particular institutional affiliations or accreditation, and/or data security infrastructure and research credentials), potential issues being present around rejection of applications (i.e. subjective perception of low-quality proposals by committees) and an increased administrative time cost for researchers related to submission requests [64]. These types of warehouses would also serve as an independent entity that manages both data collection and storage on specialized independent servers, with committees who review requests for access [65]. Whilst some warehouses with gate-keeping already exist in sport [66], they mainly provide injury data and some exposures, without other medical biomarkers or integration with data from other disciplines of sport science.

##### Federated Databases and Federated Analyses

Federated database systems are a collection of cooperating but autonomous component database systems [67]. These systems come with a series of interconnected repositories where only the results of data-related queries and analyses are available on an interfacing computer [68], thus protecting availability of data at the observation level. One example of these systems in the biosciences is called DataSHIELD [69], which facilitates epidemiological research through the collection and harmonization of data from different databases, displaying general information about data sources and embedding tools for analysis.

Federated analysis of federated database systems can be conceptualized as a two-step meta-analysis. Data are integrated and harmonized at each location (i.e. specific repository within an interconnected system), code is developed for analysis at each node, and results from these initial nodes are then aggregated through processes similar to a regular meta-analysis. Federated analyses differ from classical meta-analyses because they happen in real time and can take place without data owners being required to perform the specific analysis at each node and manually provide the results [70]. In this sense, data owners provide access for the code to be able to aggregate data, before sending this information to a central site for analysis, whilst keeping the original data in its home location. Additionally, from a statistical perspective, comparisons between analyses conducted on original merged data and federated analyses have shown similar findings for common statistical models [69, 71]. From a privacy and security perspective, all individual-level data remain protected behind the security mechanisms of a host system (housed with a local researcher or research group), and the data owner retains full control over the minimum level of aggregation required for analysis [70].

In the context of federated analyses (FA), it may also be beneficial to create a set of common study variables, also referred to as a common data model (CDM), which have a common structure and format for the variables [72–74]. The benefit of common data models is also closely related to the need for defining core outcome sets (COS), which are agreed standardized sets of outcomes that must be measured and reported, as a minimum, in all clinical trials within specific areas health or medical research [75]. Even though defining CDMs and COSs have strong importance for improving the research community’s ability to interpret, generalize and implement the research findings [76], defining these are also imperative for being able to merge different datasets whilst retaining validity over both covariates and outcomes. The capture of a COS will also facilitate collaborative research whilst simultaneously collecting additional data for local purposes and goals. Although concepts such as CDMs and COSs are discussed here within data-centric approaches, they are also foundational for enabling data reuse, meta-analyses and validation of predictive models across sport and exercise science, and connect directly with enhancing practices in a community-driven approach to collaborative science. As previously mentioned, similar standards have emerged in other disciplines (e.g., the Brain Imaging Data Structure, BIDS [42]), which illustrate how community-adopted formats can facilitate reproducibility. In sport, where protocols such as *V*O₂_max_ testing, countermovement jump assessments or standardized injury definitions are already widely used, developing equivalent shared data models could be feasible.

One example of a federated analysis is the Biobank Standardisation and Harmonisation for Research Excellence in the European Union (BioSHaRE) project, which has built a system for collaborative research and development of tools for data harmonization, database integration and federated analyses [77]. Its aim was to create a coordinated framework for harmonizing data across European population-based biobanks and to develop the technical infrastructure needed for secure, distributed analysis. Specifically, BioSHaRE sought to enable researchers to co-analyse large epidemiological datasets without moving or directly sharing individual-level data. The project successfully demonstrated these methods through the Healthy Obese Project [77], linking eight population-based cohorts across six countries to examine obesity-related risk factors using harmonized variables and distributed regression modelling.

Through workshops, teleconferences and electronic communications, participating investigators identified a core set of 96 variables for harmonization relative to specific research questions of interest. Harmonization was made possible through the use of questionnaires, standard operating procedures and data dictionaries, in addition to processing algorithms designed to connect data sources. Harmonized datasets housed in research centres across Europe were interconnected through a federated database system prior to perform any analysis. The systems developed as a part of this project place relatively few demands on the information technology infrastructure required, given that computer used for analysis can be a standard laptop/desktop running any R console that can connect to an interface module via bespoke R packages [69].

Despite the potential of federated data approaches, it is important to acknowledge considerations around their use. Responsibility for personal/individual level data is based on the real influence a specific actor has over each processing operation. This person or entity is sometimes referred to as the ‘controller’, a term that means ‘the natural or legal person, public authority, agency or other body which, alone or jointly with others, determines the purpose and means of the processing of personal data’ [70]. There may be multiple controllers for different processing operations within a federated data approach, and it is essential that roles and responsibilities for specific people are defined early. All agreements should be in writing and regulate roles and responsibilities, as well as liability for any failure to comply with the terms of the agreement [70].

In sport, a similar federated model could allow performance, injury and health data from multiple institutes or teams to be analysed collaboratively whilst respecting data ownership and privacy. By adopting shared variable definitions and harmonization protocols (analogous to those developed in BioSHaRE), the sector could conduct large-scale, cross-institutional analyses that retain local data governance yet deliver generalizable insights for athlete performance and wellbeing.

##### Synthetic Datasets

An alternative to aggregating data prior to analysis as a means of preserving privacy of individual-level observations may be to leverage the potential of synthetic data. Synthetic data refer to creating datasets that resemble the original datasets with respect to specifications of the data. For example, the synthetic dataset should be similar in sample size, number of rows for each participant and columns for each variable and data characteristics (e.g. missing values, multivariate patterns) [78]. Synthetic datasets could be merged similarly to federated analyses, but in this case, given the synthetic individual observations, classical statistical or machine learning models could be applied to the pooled synthetic observations, as performed in epidemiology [79].

Although a basic Shiny app has been recently proposed for generating synthetic data in sport (https://assetlab.shinyapps.io/SyntheticData/), creation of synthetic datasets using this application should take a careful and considered approach, given that generation of synthetic dataset is not without its caveats and presents challenges [80]. Firstly, depending on the synthetic data generation process, it may still be possible to identify specific participants, particularly given that there is likely to be a trade-off between the utility of synthetic data (i.e. how well it mirrors an original dataset) and the ability of a synthetic data to preserve the privacy of individual observations [80, 81]. Thorough revision of synthetic datasets should occur prior to releasing them openly. For some synthetic generation frameworks, synthetic variables are generated iteratively [82]. If erroneous variables are entered earlier in the iteration sequence, errors can propagate forwards as the synthetic data are constructed for subsequent variables. Within a sequential generation process, if the preceding variables are weak predictors of subsequent variables, the synthesized values will likely have low accuracy in replicating the original data, and synthesis errors will propagate, and potentially be amplified, through the chain [83]. Finally, as alluded to by Snoke et al. [84], inferences from synthetic data will only be valid if the models that are used to synthesize the data correspond to those that can be considered as having generated the original data. This means that the specifications (i.e. predictors used) for how synthetic data were generated will predicate what can be explored within any pooled synthetic data. If misspecifications are apparent, this will likely increase the risk of obtaining biased findings in pooled synthetic data. There are a range of different ways for assessing the quality of synthetic data (with this sometimes being referred to as utility) to minimize the risk of this occurring [84]. These should always be used for testing synthetic data relative to original data prior to making any data open or pooling data into a repository.

## Challenges for Collaborative Approaches Within Sport Science and Medicine

There are challenges that sport researchers may face when either using big team approaches or data-centric approaches in prospective research. If teams conducting collaborative science in sport document the challenges experienced as well as their solutions, the research community will learn how to navigate these obstacles more successfully. It is worth bearing in mind, however, that with each of these challenges come possible solutions that can be tried and tested to improve the feasibility of collaborative science approaches in practice.

*Perception of competitive advantage*: In any high-performance sport environment, whether Olympic/Paralympic or professional sport, there is a risk that no data collection will be possible for specific research questions or data contexts, due to the perception of competitive advantage of the information. This perception may also be true for synthetic replicates of the information, or summary information fed through to any federated research design, given that this is still a form of player or athlete information. As mentioned in [85], a barrier to collaboration is a desire to maintain a competitive advantage; a team’s proprietary information is often perceived as high value (and sometimes referred to as ‘trade secrets’) and is often guarded and unreleased [86]. This stems from cultural issues apparent in sport, where the pressure to win is substantial, and the culture created around the fear of losing a competitive advantage leads to excessive secrecy [64]. To navigate this, data sharing agreements could be negotiated with different teams or institutions, or federated data and synthetic data approaches could be explored to facilitate data consolidation, without the raw observations ever being transferred or made public as a part of a research process.

*Ownership and organizational control of data*: Data collected on athletes in high-performance sport environments will be subject to potentially different laws regarding ownership and perception of control over the data. Although GDPR in Europe predicates ownership of data at the athlete level for European athletes, this legislation does not hold in other countries, with specific agencies or professional leagues having more control over what is done with data. This poses a significant risk to the sustainability of any collaborative initiative longer term, given the frequent changes in executive level management within sport and the different perspectives on use of data with these changes. Furthermore, the potential commodification of athletes’ data raises debates and challenges over data ownership and related legal aspects, making data sharing even more challenging [68]. Possible solutions could look at including athletes (and athlete associations) as stakeholders on collaborative projects, so that any processes around data use for research purposes are done so with the best interests of the athlete community.

*Lack of necessary expertise*: Despite positive intentions, if any collaborative initiative is started without the requisite skills for performing quality science, mistakes that take place in smaller team research will likely propagate on a larger level in collaborative initiatives. Although this is not a problem specific to sport, it is likely a large risk for sport. Data sharing takes a commitment from the sporting scientific, university, leagues and governing body communities and grant funding opportunities to make this feasible, useful and actionable. There is little training for practitioners or faculty in data governance, management, operations and data science skills [68, 87]. A potential solution would be required training programs prior to entry into a collaborative science community. In addition, training could start at the postgraduate student level, so that collaborative science approaches form a part of industry-based doctoral programs in sport, where most applied high performance sport questions are likely to be answered. To move past this, sport should continue to better connect with statistical, data science and methodological communities to help diversify expertise as a part of these projects [29].

*Unpredictability of sport*: The unpredictability of high-performance sport, in terms of the demands placed on athletes, changing schedules, international travel, unexpected injuries, changes in plans based on coaching recommendations and rule changes that underpin different playing formats, threatens the sustainability and quality of any collaborative initiative. Care and consideration are required to align initiatives with the competition calendar to minimize difficult periods of data collection (e.g. avoid Olympic or Paralympic year within a 4-year cycle). Additionally, communication around the potential risk of compromised data collection (e.g. missing data) should happen early, and with the right statistical expertise sought.

*Infrastructure and administration ownership*: Within high-performance sport more specifically, does the responsibility sit within an academic community or within sporting institutes, government agencies or professional leagues? The answer is dependent on the specific research context and should incorporate existing governance and administration structures (i.e. organizations and federations). Infrastructure also needs to be considered, inclusive of everything from system design and architecture for housing data, standards applied to data collection processes, analytical goals and intentions for the data and methods of reporting outcomes. Similarly, costs of any infrastructure should be weighed against the wasted resources associated with underpowered studies and the inability to generalize results to specific sports.

*Collaboration*: A further challenge for collaborative approaches in sport science and medicine is the lack of coordinated priority-setting across institutions, funding bodies and professional organizations. Unlike fields such as particle physics, where collective roadmaps such as the *European Strategy for Particle Physics* [88] guide investment and research direction, sport science remains fragmented across universities, national institutes and commercial entities. This absence of alignment makes it difficult to identify shared research questions, pool funding or resolve competing methodological paradigms.

## The Benefits of Collaborative Science for Sport

Despite there being many challenges associated with building collaborative approaches for improving scientific quality, it is important to emphasize the distinct potential benefits of such approaches for sport research.

- Better informed judgements for groups that have either very low samples (i.e. paralympic and elite world class) or samples that may be difficult to access due to organizational or league related restrictions.
- Better understanding of whether effects generalize across factors of interest, inclusive of (but not limited to) coaching environments, geographical locations, other socio-demographic characteristics, athlete training and injury histories and pathway trajectories into sport.
- Better understanding of why concepts may not generalize for specific sub-groups in high-performance sport populations. For example, in paralympic sport, generalizing across class and impairment type may be difficult due to the variability within classes. Collaborative approaches have the potential to uncover sub-groups that are different, and this can be used to frame new hypotheses and exploratory research to understand why this may be the case.
- Collaboration can also progress science generally in sport by assisting in resolving longstanding disagreements, prioritizing research lines within sport and building a culture of ‘team players’ within the sport researcher and scientist eco-system [89].

## Conclusions

Sport science has suffered from issues related to smaller sample sizes, poor generalization of outcomes and difficulty performing science, particularly in high-performance sport contexts and environments. Collaborative research initiatives such as big team science and data-centric approaches to collaborative science are possible vehicles for bettering science in these contexts. Sport poses some important challenges particularly around the unpredictability of working with athletes, ownership and control of data, perceptions of competitive advantage and lack of expertise in running these initiatives. This paper serves as a first step towards better understanding and framing how collaborative approaches could help improve sport research. Regardless, there is a significant cost if we do not find and implement solutions to the challenges above, given that research conducted with underpowered and poorly conducted experimental designs will contribute to research waste, making the evidence gathered from experiments practically unusable for practitioners in the field.

## Supplementary Information

Below is the link to the electronic supplementary material.

Supplementary file1 (DOCX 28 kb)

## Footnotes

1 https://www.iso.org/standard/62085.html

## Declarations

### Data Availability

No data were used in this project.
