# Supplementary material for: Unifying to Advance Understanding: Collaborative, Community-Driven and ‘Open’ Approaches for Better Science in Sport
Source: Sports Med. 2026 Mar 16;56(4):845–59. doi: 10.1007/s40279-026-02394-8 (PMC13124818; doi:10.1007/s40279-026-02394-8)
Supplement: Supplementary file 1 — Supplementary file1 (DOCX 28 kb) [file 40279_2026_2394_MOESM1_ESM.docx]

**Supplementary 1 (S1)**

**Table 1**. Examples of big team science in other fields.

| **Name** | **Characteristics** | **Focus** | **Additional information** |
| --- | --- | --- | --- |
| ***Psychological Science Accelerator*** (PSA) | The PSA is a globally distributed network of psychological science laboratories consisting of 2468 members across 73 countries. | The PSA’s mission is to accelerate the accumulation of reliable and generalizable evidence in psychological science and research.  The PSA coordinates data collection for studies that are selected democratically within the big team. These studies are often centred on the improvement of diversity in psychology samples (the PSA can be found here: <https://psysciacc.org/> and has been published at [26]). | The PSA is an exemplar of how a big team approach is more than just a multi-centre approach to conducting research, in the sense that it is ongoing rather than being anchored in a discrete project.  The PSA has developed comprehensive administration structures, documentation and repeatable processes, which can be leveraged for new projects undertaken by the community. The PSA has developed policies and protocols that facilitated creating and governing a collaborative research community that is functional and sustainable [90], so that new members of the community can understand the logic and process that sits behind decision-making within the PSA. |
| ***Disturbance and Resources Across Global Grasslands*** (DRAGNet) (and ***The Nutrient Network***) | The Nutrient Network is a research network consisting of 130+ grassland sites worldwide (see: <https://nutnet.org/home>).  The Nutrient Network has sparked additional big team initiatives such as the Disturbance and Resources Across Global Grasslands Network (DragNet) (<https://nutnet.org/dragnet>) [91]. | DragNet assesses the generalization across and influence of site/location on factors influencing disturbance recovery and community assembly in herbaceous dominated ecosystems.  [91]. | The infrastructure developed within DRAGNet permits the pooling of data across a variety of temporal scales, facilitating longitudinal analyses [92] to identify effects across multiple time scales and settings, providing a baseline set of predictors for restoration success [91]. |
| ***ManyPrimates*** | ManyPrimates was launched in 2018. It involves more than 150 researchers ( <https://manyprimates.github.io/>).  This informal organization of researchers focusses on 41 primate species. | The ManyPrimates project was initiated to facilitate collaboration across study sites in primate cognition research. ManyPrimates is focussed on closely related primate species, given that cross-species characteristics related to phylogeny are important for research in primate cognitive evolution [28,93]. | *ManyPrimates* was initiated to facilitate collaboration across study sites in primate cognition research. *ManyPrimates* was initially piloted through a case study investigating primate short-term memory. In this initial study, 176 primate participants from 12 primate species housed at 11 sites across Africa, Asia, North America and Europe were included, and all subjects were tested in a delayed-response task using a consistent methodology [28].  The pilot established infrastructure for future studies, which facilitated researcher communication and coordination of activities, developed policies and protocols and provided a platform for attracting future members [28]. |
| ***ManyBabies*** | ManyBabies was launched in 2015, and now involves 450+ researchers (with their collective presence also established on GitHub: <https://manybabies.github.io/>, and also through the open science framework <https://osf.io/rpw6d/>).  There are data from 2329 infants collected by 150 researchers in 16 countries across the world [27]. | ManyBabies is a collaborative initiative for improving best scientific practices in developmental psychology research for research related to infants. Together, the researchers address difficult theoretical and methodological questions regarding infant early development [94]. | ManyBabies does not engage in direct replication efforts, where a study is chosen from the literature and replicated exactly. Rather, ManyBabies focusses on testing key theoretical claims through design of the best possible test of a claim (i.e. conceptual replication) [43].  Sources of variation in effects are tested across laboratories, methods and populations, assessing the generalizability and robustness of outcomes across different contexts. |
